# Supplementary material for: Durvalumab after Sequential High Dose Chemoradiotherapy versus Standard of Care (SoC) for Stage III NSCLC: A Bi-Centric Trospective Comparison Focusing on Pulmonary Toxicity
Source: Cancers (Basel). 2022 Jun 30;14(13):3226. doi: 10.3390/cancers14133226 (PMC9265119; doi:10.3390/cancers14133226)
Supplement: Supplementary file 1 [file cancers-14-03226-s001.zip › cancers-1764639-supplementary.pdf]

Supplementary Materials: Durvalumab after Sequential High-Dose Chemoradiotherapy versus Standard of Care (SoC) for Stage III NSCLC: A Bi-Centric Retrospective Comparison Focusing on Pulmonary Toxicity

Romana Wass, Maximilian Hochmair, Bernhard Kaiser, Brane Grambozov, Petra Feuerstein, Gertraud Weiß, Raphaela Moosbrugger, Felix Sedlmayer, Bernd Lamprecht, Michael Studnicka and Franz Zehentmayr

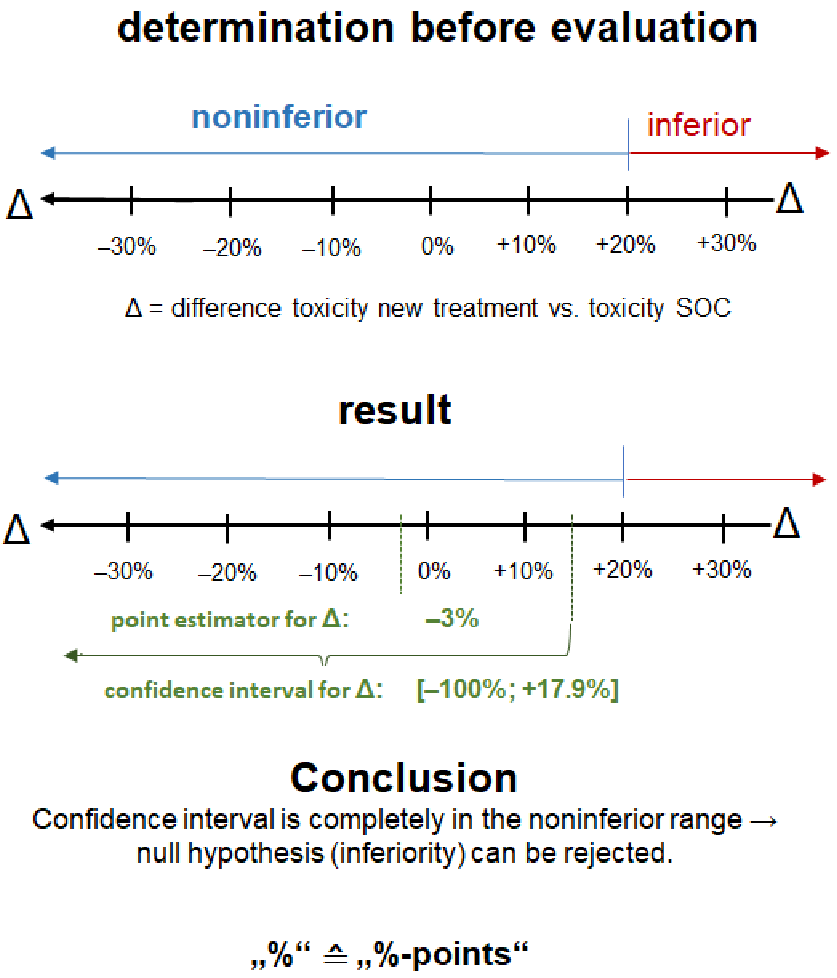

Figure S1. Non-inferiority trials test, whether an experimental intervention is as efficacious as standard treatment. Therefore, it is necessary to define a cut-off below which the experimental arm is regarded as "non-inferior". In the current study, this value was set at +20% meaning that if the high-dose group had 20% or less excess pulmonary toxicity compared to standard treatment, it could be accepted as non-inferior. Hence, the one-sided 95% confidence interval may not include the range of 20% or higher.

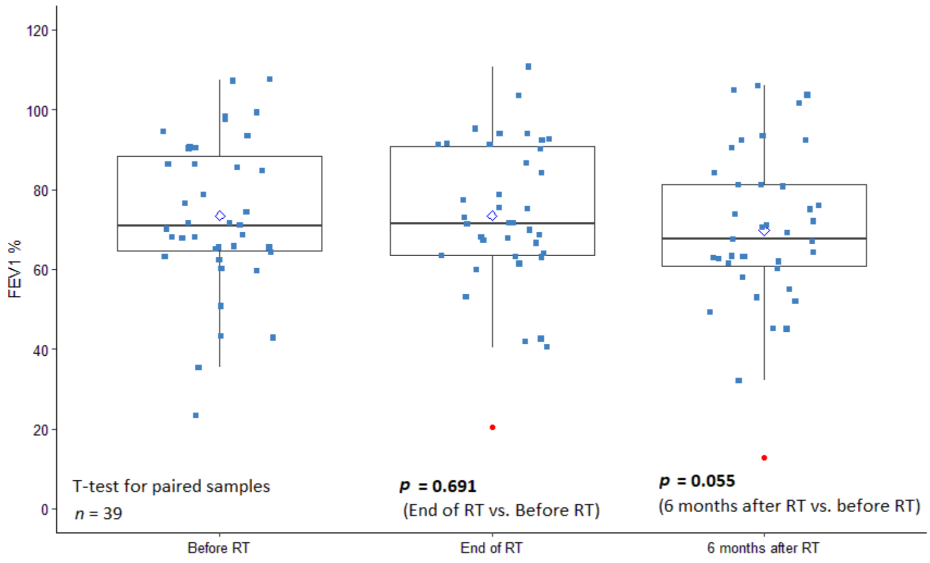

Figure S2. In the high-dose cohort, moderate changes in FEV1 could be observed at six months after RT compared to baseline (=before RT): *n* = 39, *T*-test for paired samples.

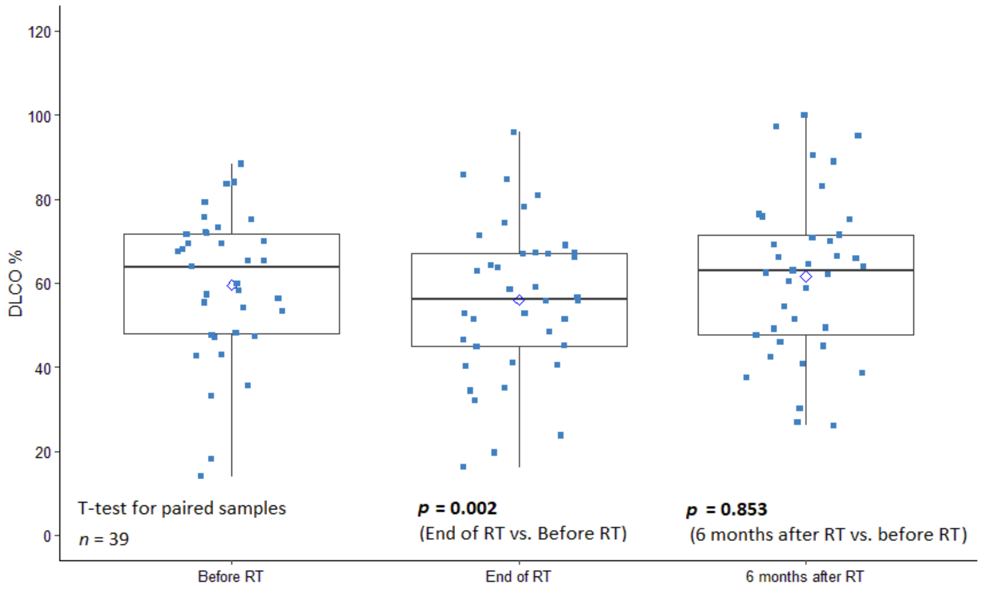

Figure S3. In the high-dose cohort, moderate changes in DLCO could be observed. The statistically significant decrease at the end of RT compared to baseline (=before RT) recovered six months later: *n* = 39, *T*-test for paired samples.

Table S1. Patient match.

Patient Match: High Dose Patients = Red, SoC Patients Green						
Patient No.	Salzburg = 1, Vienna = 0	Age	Sex: Male = 1, Female = 0	0 = ECOG 0 or 1, 1 = ECOG 2 or Higher	Smoking Status: 1 = ex- or Current Smoker, 0 = Never Smoker	Histology: 1 = SCC, 2 = AC, 3 = NOS
1	1	62.7	0	0	1	2
31	0	52.79	0	0	1	2
11	0	56.35	0	0	1	2
2	1	59.3	0	0	1	1
17	0	56.96	0	0	1	1
14	0	59.83	0	0	1	1
4	0	49.92	0	0	1	1
3	1	61.1	0	0	1	2
5	0	57.01	0	0	1	2
8	0	53.3	0	0	1	2
27	0	5514	0	0	1	2
33	0	57.96	0	1	1	2
4	1	71.93	0	0	1	2
6	0	65.01	0	0	1	2
5	1	73.98	0	0	1	1
25	0	72.9	0	0	1	2
6	1	69.37	0	0	1	1
13	0	59.35	0	0	1	1
10	0	74.71	0	0	1	1
7	1	72.93	0	0	1	2
2	0	79.47	0	0	1	2
8	1	69.73	0	0	0	1
9	1	69.21	0	0	1	2
3	0	66.51	0	0	1	2
10	1	72.54	0	0	1	2
28	0	74.62	0	0	1	2
11	1	73.68	0	0	0	2
12	0	80.76	0	0	0	3
12	1	70.09	0	0	1	2
18	0	69.1	0	0	1	2
13	1	64.95	1	0	1	1
14	1	63.6	1	0	1	1
7	0	63.17	1	0	1	1
15	1	67.09	1	0	1	2
1	0	75.74	1	0	1	2
16	1	77.22	1	0	0	2
17	1	70.98	1	0	1	2
19	0	62.72	1	0	1	2
18	1	70.56	1	0	1	1
15	0	67.09	1	0	1	1
19	1	32.61	1	0	1	2
9	0	46.54	1	0	1	1
20	1	68.74	1	0	1	1
16	0	62.81	1	0	1	1

21	1	59.06	1	0	1	1
34	0	52.6	1	0	1	1
22	1	66.08	1	0	1	1
20	0	65.91	1	0	1	1
23	1	70.24	1	0	1	2
21	0	69.16	1	0	1	2
24	1	63.16	1	0	1	1
23	0	66.05	1	0	1	3
25	1	58.89	1	0	1	2
26	1	79.54	1	0	1	1
22	0	76.87	1	0	1	1
27	1	52.63	1	0	0	2
28	1	71.00	1	0	1	1
29	1	73.11	1	0	1	1
29	0	71.09	1	0	1	1
30	1	72.73	1	0	1	2
31	1	56.86	1	0	1	2
24	0	61.71	1	0	1	2
32	1	62.79	1	0	1	1
30	0	66.83	1	0	1	1
33	1	83.41	1	0	1	2
34	1	50.97	1	0	1	2
32	0	46.67	1	0	1	3
35	1	56.2	1	0	1	2
35	0	60.52	1	0	1	2
36	1	63.23	1	0	1	2
26	0	62.92	1	0	1	2
37	1	72.79	1	0	0	2
38	1	68.01	1	0	1	2
39	1	57.54	1	0	1	2
40	1	66.83	1	0	1	2
41	1	59.89	1	0	1	1
36	0	56.03	1	0	1	1
42	1	62.09	1	0	1	2
